# Experimental composable key distribution using discrete-modulated continuous variable quantum cryptography

**DOI:** 10.1038/s41377-025-01924-9

**Published:** 2025-07-28

**Authors:** Adnan A. E. Hajomer, Florian Kanitschar, Nitin Jain, Michael Hentschel, Runjia Zhang, Norbert Lütkenhaus, Ulrik L. Andersen, Christoph Pacher, Tobias Gehring

**Affiliations:** 1 Center for Macroscopic Quantum States (bigQ), Department of Physics, Technical University of Denmark, 2800 Kongens Lyngby, Denmark; 2 Vienna Center for Quantum Science and Technology (VCQ), Atominstitut, Technische Universität Wien, Stadionallee 2, 1020 Vienna, Austria; 3 AIT Austrian Institute of Technology, Center for Digital Safety & Security, Giefinggasse 4, 1210 Vienna, Austria; 4 Institute for Quantum Computing and Department of Physics and Astronomy, University of Waterloo, Waterloo, ON N2L 3G1 Canada; 5 FragmentiX Storage Solutions GmbH, Wohllebengasse 10/7, 1040 Vienna, Austria

**Keywords:** Quantum optics, Single photons and quantum effects

## Abstract

Establishing secure data communication necessitates secure key exchange over a public channel. Quantum key distribution (QKD), which leverages the principles of quantum physics, can achieve this with information-theoretic security. The discrete modulated (DM) continuous variable (CV) QKD protocol, in particular, is a suitable candidate for large-scale deployment of quantum-safe communication due to its simplicity and compatibility with standard high-speed telecommunication technology. Here, we present the first experimental demonstration of a four-state DM CVQKD system, successfully generating composable finite-size keys, secure against collective attacks over a 20 km fiber channel with 2.3 × 10^9^ coherent quantum states, achieving a positive composable key rate of 11.04 × 10^−3^ bits/symbol. This accomplishment is enabled by using an advanced security proof, meticulously selecting its parameters, and the fast, stable operation of the system. Our results mark a significant step toward the large-scale deployment of practical, high-performance, cost-effective, and highly secure quantum key distribution networks using standard telecommunication components.

## Abstract (web summary)

We demonstrate the first four-state DM CVQKD system generating composable secure keys over 20 km fiber, marking progress toward practical, high-performance, cost-effective, and highly secure QKD networks using standard telecommunication components.

## Graphical abstract

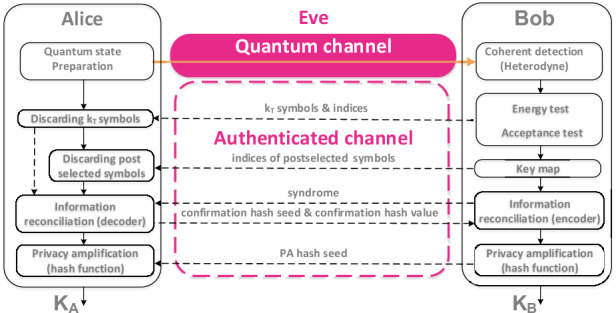

## Introduction

Quantum key distribution (QKD)^1,2^ has emerged as a pivotal technology for secure communication, leveraging the principles of quantum mechanics to enable information-theoretic secure key exchange between two (or more) distant parties. Among the various approaches to QKD, continuous variable (CV) QKD is particularly notable for its compatibility with standard telecom technologies, allowing room temperature operation and high-rate secure key distribution^3–5^ over metropolitan distances compared to discrete-variable QKD^6,7^ which currently facilitates key exchange over higher channel attenuations than CVQKD. This compatibility also facilitates miniaturization through photonic integration^3,8^, and allows seamless integration with current telecom networks^9–11^.

In the realm of CVQKD, Gaussian modulated protocols^12–15^ have traditionally dominated the field. These protocols use coherent states with Gaussian-distributed quadratures to encode key information, have fairly advanced security proofs^16,17^, and feature in all of the long-distance-record experiments for CVQKD (see Table 2 in ref. ^18^). Despite these advantages, Gaussian-modulated CVQKD protocols face significant implementation challenges. One major issue is the need for a large constellation of states to accurately approximate the continuous Gaussian distribution assumed in ideal security proofs. This requirement necessitates a high bit resolution for the digital-to-analog converter (DAC), which not only limits system speed but also complicates the integration of practical, coherent telecommunication components. This complexity makes it significantly harder to implement fast error-correction routines, thus becoming a major bottleneck preventing real-time execution of the complete protocol. Additionally, it also places a high demand on the rate from the quantum random number generator. Finally, even with a good approximation of continuous Gaussian modulation (through a large number of states in the constellation^19^), a complete security analysis must account for the impact of discretization^20^.

Discrete-modulated (DM) CVQKD^21–23^ addresses these issues directly by using a finite set of quantum states, such as those from a quadrature phase shift keying (QPSK) alphabet. This approach simplifies the system implementation and makes it more accessible for real-world applications. Recent advancements in security analysis^24–26^ have provided strong theoretical foundations for the composable finite-size security^27–30^ of DM CVQKD. However, there has been a lack of experimental demonstrations validating the practical viability of distributing composable secure keys using DM CVQKD.

In this article, we report the first experimental demonstration of DM CVQKD with composable finite-size security against collective attacks over a 20 km fiber channel. We achieved this using a CVQKD system implementing standard QPSK modulation and deploying an advanced composable finite-size security proof^28^. By carefully optimizing the system and ensuring high stability and high-speed operation, we achieved a positive composable key fraction of 11.04 × 10^−3^ bits/symbol using a total of *N* ≈ 2.3 × 10^9^ coherent quantum states with a security parameter of *ϵ* = 1 × 10^−10^. After implementing the full protocol stack, including classical error-correction and privacy amplification, we obtained 25.94 Mbit of key material that, upon acceptance, is composably secure against independent, identically distributed (i.i.d.) collective attacks and ready for cryptographic tasks.

## Results

### DM CVQKD protocols and composable secure key

#### Protocol description

**Fig. 1:**
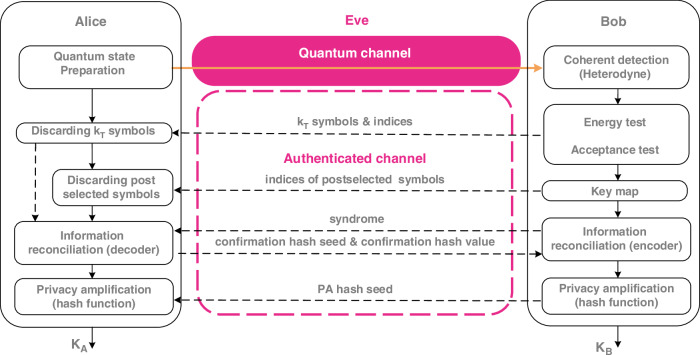
**Discrete modulated continuous variable quantum key distribution protocol with composable security**. See the main text for the details

Figure 1 shows a flowchart of the prepare-and-measure DM CVQKD protocol with QPSK alphabet. The protocol steps read as follows:

1. ***State Preparation—*** Alice, using a random number generator, prepares one out of four coherent states $\alpha$ with $\alpha \in \{| \alpha | {e}^{\frac{i\pi }{4}},| \alpha | {e}^{\frac{3i\pi }{4}},| \alpha | {e}^{\frac{5i\pi }{4}},| \alpha | {e}^{\frac{7i\pi }{4}}\}$ according to a uniform distribution and sends it to Bob via the quantum channel controlled by Eve while keeping a record of the sent state in her classical register. We denote this classical two-bit register by *x*_*j*_.
2. ***State Measurement—*** Bob performs a heterodyne measurement on the received quantum state and determines the quadratures *q* and *p*, which he stores as a complex number *y*_*j*_ in his classical register.

Steps 1 and 2 are repeated *N* times.

3. ***Energy Test—*** Once the quantum phase of the protocol is completed, Bob performs an Energy Test (see Supplementary Information S1) on *k*_*T*_ < *N* randomly chosen symbols. The Energy Test (see Theorem 2 in ref. ^28^) is an integral part of the security analysis that allows us to consider finite-dimensional Hilbert spaces, while still keeping a rigorous security statement. In case the Energy Test is passed, most of the weight of the received signals lies within a finite-dimensional Hilbert space, except with some small probability *ϵ*_ET_. If the energy test is not passed, the protocol aborts.
4. ***Acceptance Test—*** Alice discloses the data used for the Energy Test and Bob uses this information to determine statistical estimators for the observables used (see Supplementary Information S1). In case the Acceptance Test (see Theorem 3 in ref. ^28^) is passed, the observed quantum states lie within the acceptance set, except with some small probability *ϵ*_AT_. If they do not lie within a predetermined acceptance set, the protocol aborts.
5. ***Key Map—*** To determine a key string $\bar{z}$, Bob applies a key map on the remaining *n*: = *N* − *k*_*T*_ symbols: he discretizes his measurement outcomes to elements in the set {0, 1, 2, 3, ⊥}, where discarded symbols are mapped to ⊥, allowing for postselection (see refs. ^25,31^ for details). The corresponding key map performed on each of the symbols reads
  1 $${z}_{j}({y}_{j})\!:=\left\{\begin{array}{ll}0\,{\text{if}}\,\,0\le \arg ({y}_{j}) < \frac{\pi }{2}&\wedge \,{\Delta }_{r}\le | {y}_{j}| \le M,\\ 1\,\text{if}\,\,\frac{\pi }{2}\le \arg ({y}_{j}) < \pi &\wedge \,{\Delta }_{r}\le | {y}_{j}| \le M,\\ 2\,\text{if}\,\,\pi \le \arg ({y}_{j}) < \frac{3\pi }{2}&\wedge \,{\Delta }_{r}\le | {y}_{j}| \le M,\\ 3\,\text{if}\,\,\frac{3\pi }{2}\le \arg ({y}_{j}) < 2\pi &\wedge \,{\Delta }_{r}\le | {y}_{j}| \le M,\\ \perp &{\rm{otherwise},}\\ \end{array}\right.$$
  where Δ_*r*_ and *M* are postselection parameters (see the Security Argument section for the meaning of *M*).
6. ***Reverse Reconciliation—*** Information reconciliation is done in multiple sub-blocks, partitioning the remaining symbols to match the block size of the error correcting code. For each such sub-block Bob uses the classical authenticated channel to send the syndrome of an error correcting code to Alice who corrects her key string $\bar{x}$.
  This is followed by Error Verification (sometimes called Confirmation): Alice and Bob compare hash-values of their key strings which they calculate using a randomly chosen hash function from a family of universal hash functions to confirm that all errors have been corrected successfully. Except with probability *ϵ*_EC_ they share an identical string afterwards.
7. ***Privacy Amplification—*** Finally, Alice and Bob turn their bit string into the secure key by applying a randomly chosen hash function from a universal family. Except with some small probability *ϵ*_PA_ they obtain a secret key.

#### Application of the security argument

In this work, we applied the composable security proof described in detail in refs. ^28,32^. In the following, we give an overview of the idea and its application to the present experiment.

Ultimately, we want to use the generated secure key in larger cryptographic tasks. Thus, we aim for so-called composable security^33,34^, which quantifies security by a parameter *ϵ* > 0 representing Eve’s advantage in distinguishing the real key from an ideal key. Note that the protocol is trivially secure if it aborts.

According to the setting of QKD, the quantum channel is under the control of Eve. Thus, we cannot assume a priori that the maximum photon number of the received quantum optical signals is bounded. We tackle this problem by performing an Energy Test, where Bob discloses *k*_*T*_ randomly chosen symbols via the classical channel and analyzes his measurements in those symbols according to Theorem 2 in ref. ^28^: First, he picks a weight *w* ∈ [0, 1], a photon cutoff number *n*_*c*_, a testing parameter *β*_test_ > 0 and a number of allowed outliers *ℓ*_*T*_. Then, he counts the number of rounds in which the measurement results *y*_*k*_ lie outside a circle with radius *β*_test_ in the phase space. In case this count exceeds *ℓ*_*T*_, Bob aborts the protocol. The test is designed to fail except with some small probability *ϵ*_ET_ for states with a weight larger than *w* outside the cutoff space. Then, based on the disclosed measurement results, Alice and Bob determine statistical estimators for their considered observables and check if they lie within a pre-defined acceptance set. We designed a so-called ‘Non-unique-acceptance test’, which allows the acceptance of a continuum of statistics, making the test more noise-robust. For each observable *X*, we quantify this extension by a parameter *t*_*X*_: = *t*_*F*_*μ*_*X*_, where *t*_*F*_ is introduced for convenience to measure *t*_*X*_ in multiples of the error bound *μ*_*X*_. Then, except with probability *ϵ*_AT_, the test aborts on states outside this set, which allows restricting the analysis on states within the set. Note that the separation into Energy Test and Acceptance Test is somewhat didactic: they can be combined into one single statistical test, giving rise to a set ${{\mathcal{S}}}^{{\rm{E}}\&{\rm{A}}}$.

Finally, to handle unbounded observables, we introduce a finite detection region in phase space, parametrized by *M* > 0 that is smaller or equal to the detection range of the physical detector employed in our experiment: ${\mathcal{M}}:=\{\gamma \in {\mathbb{C}}:\,| \gamma | < M\}$.

The purely classical protocol steps of error-correction and privacy amplification (Steps 6 and 7) enter the security argument in the form of security parameters *ϵ*_EC_, *ϵ*_PA_ and a leakage parameter ${\delta }_{{\rm{leak}}}^{{\rm{EC}}}$. The latter is obtained directly from the practical setup, as will be explained later. In this work, we aim for security against i.i.d. collective attacks where Eve is assumed to prepare ancilla states which may interact with each protocol round in an identical way and henceforth are stored in Eve’s quantum memory until Alice and Bob have finished executing their protocol. This leads to the following security statement.

##### Theorem 1

(**Security against i.i.d. collective attacks**^28^) Let ${{\mathcal{H}}}_{A}$ and ${{\mathcal{H}}}_{B}$ be separable Hilbert spaces and let ${\epsilon }_{{\rm{ET}}},{\epsilon }_{{\rm{AT}}},\bar{\epsilon },{\epsilon }_{{\rm{EC}}},{\epsilon }_{{\rm{PA}}} > 0$. The objective QKD protocol is ${\epsilon }_{{\rm{EC}}}+\max \left\{\frac{1}{2}{\epsilon }_{{\rm{PA}}}+\bar{\epsilon },{\epsilon }_{{\rm{ET}}}+{\epsilon }_{{\rm{AT}}}\right\}$-secure against i.i.d. collective attacks, given that, in case the protocol does not abort, the secure key length *ℓ* is chosen to satisfy

2 $$\begin{array}{ll}\frac{\ell }{N}\le\frac{n}{N}\left[\mathop{\min }\limits_{\rho \in {{\mathcal{S}}}^{{\rm{E}}\&{\rm{A}}}}H{(X| {E}^{{\prime} })}_{\rho }-\Delta (w)-\delta (\bar{\epsilon })\right]\\\qquad-{\delta }_{{\rm{leak}}}^{{\rm{EC}}}-\frac{2}{N}{\log }_{2}\left(\frac{1}{{\epsilon }_{{\rm{PA}}}}\right),\end{array}$$

where ${\delta }_{{\rm{leak}}}^{{\rm{EC}}}$ takes the classical error correction cost into account, $\Delta (w):=\sqrt{w}{\log }_{2}(| Z| )+(1+\sqrt{w})h\left(\frac{\sqrt{w}}{1+\sqrt{w}}\right)$, $\delta (\bar{\epsilon }):=2{\log }_{2}\left({\rm{rank}}({\rho }_{X})+3\right)\sqrt{\frac{{\log }_{2}\left(2/\bar{\epsilon }\right)}{n}}$, ${{\mathcal{S}}}^{{\rm{E}}\&{\rm{A}}}$ contains all states that pass both the Energy Test and the Acceptance Test except with probability *ϵ*_ET_ + *ϵ*_AT_ and *n*: = *N* − *k*_*T*_.

By $H{(X| {E}^{{\prime} })}_{\rho }$, we mean the conditional von Neumann entropy; by ∣*Z*∣, we denote the number of different key map elements that are not discarded during post-selection, which for the present protocol is 4 and $\bar{\epsilon }$ is a smoothing parameter that appears in the security argument (see ref. ^28^ for details).

The numerical security arguments require finite-dimensional spaces for the evaluation of the key rate formula in Eq. (2). Thus, we use the finite cutoff as well as the weight that was introduced for the energy test and employ the dimension-reduction method^35^ (using the improved correction term from ref. ^36^) which relates the infinite-dimensional evaluation to a finite-dimensional cutoff representation at the cost of introducing a correction term Δ(*w*). We will discuss the choice of *w* and its interplay with other parameters in the experimental demonstration section.

For the remaining task of solving the optimization problem in Eq. (2), and for details regarding the application of the security argument, we refer the reader to Supplementary Information S1.

### System implementation and post-processing

**Fig. 2:**
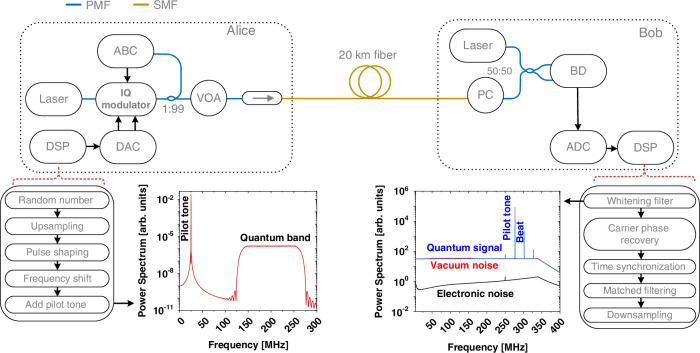
**DM CVQKD set-up**. Schematic of the quantum key distribution (QKD) system, detailing all key components and digital signal processing (DSP) modules. At Alice’s side: CW laser (continuous wave laser), IQ modulator (in-phase and quadrature modulator), VOA (variable optical attenuator), Faraday isolator (indicated by the arrow), DAC (digital-to-analog converter) and ABC (automatic bias controller). At Bob’s side: CW laser used as a local oscillator (LO), BD (balanced detector), polarization controller (PC), and ADC (analog-to-digital converter). Alice’s and Bob’s stations utilize polarization-maintaining fiber (PMF) components, while the quantum channel is a spool of standard single-mode fiber (SMF). All electronic connections are represented by black lines

In Fig. 2 we show a schematic of our CVQKD system, which uses advanced digital signal processing (DSP) and simple optical modules to facilitate secure key exchange between a sender (Alice) and a receiver (Bob) with composable security against collective attacks. We refer to this system as a digital CVQKD system^18,19,37^, as it simplifies the optical subsystem by integrating hardware functions into the DSP module (See the Materials and Methods section for a detailed description of the optical subsystem and the DSP modules).

The post-processing phase is responsible for transforming the DSP-processed measurement data into a secure key. As illustrated in Fig. 1, this phase encompasses several tasks, already defined and described in the protocol description subsection. These tasks require communication over a classical channel, which must be error-free and authenticated to prevent man-in-the-middle attacks.

#### Message authentication

Information-theoretic secure message authentication is achieved by calculating and exchanging two message tags (one for each communication direction) using a message authentication code (MAC) based on a universal polynomial hash function which is randomly selected. For each key block these two message tags are calculated once for the concatenation of all messages exchanged between Alice and Bob. The MAC uses a constant short (96 bits) pre-shared key for the polynomial evaluation, and a one-time pad which is replenished with secure quantum keys at a cost of 96 bits per key block.

#### Energy test and acceptance test

The first step, Energy and Acceptance Tests, involves disclosing *k*_*T*_ symbols. We performed the security analysis for three different values of *k*_*T*_, i.e., 0.4 × *N*, 0.45 × *N* and 0.5 × *N* (recall *N* ≈ 2.3 × 10^9^). After these tests, the disclosed symbols were used to estimate parameters, such as the signal-to-noise ratio (SNR), which are critical for subsequent tasks. Afterwards, the disclosed symbols were discarded.

#### Key mapping and post selection

To implement key mapping on Bob’s measurements, radial post selection was employed with the parameter *M* set to 3.889 natural units (NU) (see Supplementary Information S2 for the definition of NU), while Δ_*r*_ was chosen within the range of 0.3 to 0.7 NU. These parameters are vital for the error-correction step, as they allow control over the SNR by discarding data below Δ_*r*_ and above *M*. The key mapping step concludes with the disclosure of a fraction *r*_⊥_ of the discarded symbols.

#### Reverse reconciliation

Following key mapping, Alice and Bob perform reverse reconciliation using low-density parity-check (LDPC) codes, which operate close to the Shannon limit at low SNRs. We have created a collection of LDPC codes with a constant block size of 512,000 bits for the binary symmetric channel (BSC) and code-rates adapted to the relevant range of SNRs (see Table 1).

**Table 1:** Effect of post-selection on error-correction with test ratio 40%

Δ_*r*_, NU	*r*_⊥_, %	SNR	*R*, %	FER, %	EC_leak_, bits/symbol
0	0	0.0944	3.5	0.018	1.9298
0.30	7.70	0.1027	4	0.020	1.7721
0.35	10.33	0.1057	4.5	0.445	1.7124
0.40	13.28	0.1091	4.5	0.021	1.6562
0.45	16.50	0.1130	5	3.997	1.5866
0.50	19.96	0.1174	5	0.045	1.5207
0.55	23.62	0.1222	5	0.024	1.4511
0.60	27.43	0.1275	5.6	0.075	1.3700
0.65	31.37	0.1332	6	3.462	1.2907
0.70	35.37	0.1394	6	0.028	1.2149

Post-selection parameter: Δ_*r*_, fraction of discarded symbols due to post-selection: *r*_⊥_, signal-to-noise ratio: SNR, code rate: *R*, frame error rate: FER, leakage: EC_leak_

Alice and Bob divide their string of key-mapped data symbols into blocks matching the fixed block size of the LDPC codes. The LDPC code with the highest threshold below the estimated SNR value is selected for correction. Using the parity check matrix of the selected LDPC code, Bob calculates and sends the syndrome of each block to Alice, who corrects her data block. We set the maximum number of LDPC decoder iterations to 200, which gives a good compromise between FER achieved and run-time. Our throughput-optimized decoder converges typically in 20–100 decoder iterations, taking 1 ms per iteration on a single core of a 2.8 GHz AMD EPYC 7402P CPU. This corresponds to a throughput of roughly 5 × 10^6^ to 25 × 10^6^ corrected bits/s.

We characterize each LDPC code by its code rate $R=\frac{{L}_{{\rm{LDPC}}}-{L}_{{\rm{syn}}}}{{L}_{{\rm{LDPC}}}}$, with the block length *L*_LDPC_ and the syndrome length *L*_syn_, and its SNR-threshold which we define to be the SNR where the frame error rate FER = 0.5 using a maximum of 200 decoder iterations. Success or failure of the error correction is determined by the decoder algorithm itself and the subsequent error verification (see Error Verification subsection). The information leakage EC_leak_ due to error-correction is calculated based on the number of corrected blocks *B*_cor_ and the number of failed blocks *B*_fail_:

3 $$\begin{array}{ll}{{\rm{EC}}}_{{\rm{leak}}}=\frac{{B}_{{\rm{cor}}}}{{B}_{{\rm{cor}}}+{B}_{{\rm{fail}}}}\times 2\left(1-R\right)\\\qquad\qquad+\,\frac{{B}_{{\rm{fail}}}}{{B}_{{\rm{cor}}}+{B}_{{\rm{fail}}}}\times \left({\rm{QRE}}-\Delta (w)-\delta (\bar{\epsilon })\right),\end{array}$$

where ${\rm{QRE}}:=\mathop{\min }\nolimits_{\rho \in {{\mathcal{S}}}^{{\rm{E}}\&{\rm{A}}}}H{(X| {E}^{{\prime} })}_{\rho }$ denotes the entropy term in Eq. (2), Δ(*w*) is a weight correction term, $\delta (\bar{\epsilon })$ is the correction due to applying the asymptotic equipartition property of the complete input block after post-selection, and it can be related to ${\delta }_{{\rm{leak}}}^{{\rm{EC}}}$ by scaling with $\frac{n}{N}$. The first term represents the total information content in the syndromes of all corrected blocks, while the second term accounts for the complete information of all failed blocks. Even when a failed block is fully disclosed, the information leakage cannot exceed the block’s contained information.

**Fig. 3:**
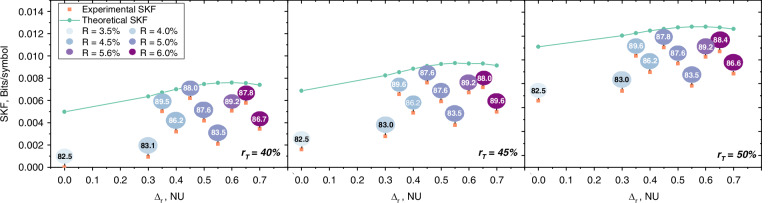
**Secure Key Fraction (SKF** **=** **ℓ/N) vs. radial postselection parameter for different testing ratios** ***r***_***T***_. The green curves represent the theoretical SKF for assumed (constant) 92% error-correction efficiency, the orange dots illustrate the secure key fraction for the experimental data. The numbers in the balloons state the average efficiency $\bar{\beta }$ of LDPC codes used for error-correction, while the color of the balloons indicates the code rate *R*

Table 1 illustrates the impact of the postselection parameter Δ_*r*_ on the SNR, the error-correction performance, and the leakage. For example, using the same LDPC code (see e.g., code *R* = 5%) with increasing Δ_*r*_ will result in a lower FER, thus reducing the leakage EC_leak_. But at the same time, due to the rising SNR, the efficiency for corrected blocks drops, so the average efficiency (Eq. (4)) still decreases in spite of lower FER.

4 $$\bar{\beta }=\frac{1}{{B}_{{\rm{tot}}}}\mathop{\sum }\limits_{k=1}^{{B}_{{\rm{tot}}}}{\beta }_{{\rm{k}}}=\frac{1}{{B}_{{\rm{tot}}}}\mathop{\sum }\limits_{k=1}^{{B}_{{\rm{tot}}}}\frac{R}{{I}_{{\rm{AB,k}}}}$$

where *B*_tot_ is the total number of blocks, *I*_AB,k_ is the mutual information for the *k*th block, and using a code rate of zero for failed (and disclosed) blocks. Moreover, due to the higher number of discarded symbols, the total secure key fraction drops (see Fig. 3). This highlights the critical trade-off between these parameters to maximize the final secure key length.

#### Error verification (Confirmation) with polynomial hashing

After error correction, Alice randomly selects a hash function from a family of polynomial universal hash functions that map from 512,000 to 96 bits and sends the function index to Bob. Then Alice and Bob use this function to calculate a hash value for each data block. Alice sends her hash value to Bob which compares Alice’s and his hash value. If the hash values are different, reconciliation has failed and Bob discloses his data block.

#### Privacy amplification

Finally, all blocks are concatenated to form one large block. Alice computes the length of the final secure key based on Eq. (2), depending on the total security parameter *ϵ* and the information leakage from error-correction.

Alice randomly selects a hash function from the family of universal Toeplitz hash functions^38^ that maps from the length of the input block to the length of the final secure key, and sends (the first row and column of) the Toeplitz matrix to Bob.

Alice and Bob each apply this hash function and obtain the secure key.

## Experimental demonstration

Before presenting the results, it is essential to discuss and specify the parameters used in this work and to comment on the application of the security argument.

Recall that the security argument behind Theorem 1 is based on comparing the observations with the honest implementation of the protocol, followed by a hard accept or abort decision. In our proof-of-concept experiment, we characterized the expected behavior in the non-adversarial scenario experimentally and chose appropriate quantities for both the Acceptance and the Energy Test. In particular, we chose *t*_*X*_ = *μ*_*X*_ (so, *t*_*F*_ = 1) in the Acceptance Testing theorem (see ref. ^28^ or Theorem 3 in Supplementary Information S1). For a full protocol run, the observed quantities must be processed as described by the tests. In case the observations pass the Energy Test and lie within the acceptance set, the terms $\mathop{\min }\nolimits_{\rho \in {{\mathcal{S}}}^{{\rm{E}}\&{\rm{A}}}}H{(X| {E}^{{\prime} })}_{\rho }-\Delta (w)$ in the security statement are equal to the pre-calculated value, otherwise the protocol aborts. Thus, the computationally expensive key rate calculation is performed beforehand, during the characterization of the system, and is no bottleneck for the protocol execution. The generalization of recently published variable-length security arguments for discrete-variable QKD^39^ to the continuous variable regime could ease application and improve key rates further.

In this work, we aim for a total composable security parameter of *ϵ* = 1 × 10^−10^. The connection between the security parameters of the sub-protocols and the total security parameter is detailed in Theorem 1. Additionally, we need to take the security parameter of the Random Number Generator as well as the message authentication into account. However, since we work in the framework of composable security, the respective security parameters can simply be added to the existing security statement from Theorem 1. Consequently, we obtain $\epsilon ={\epsilon }_{{\rm{RNG}}}+{\epsilon }_{{\rm{auth}}}+{\epsilon }_{{\rm{EC}}}+\max \left\{\frac{1}{2}{\epsilon }_{{\rm{PA}}}+\bar{\epsilon },{\epsilon }_{{\rm{ET}}}+{\epsilon }_{{\rm{AT}}}\right\}$. Our approach was to approximately balance both terms in the maximum expression for the total security parameter, setting, $\frac{1}{2}{\epsilon }_{{\rm{PA}}}+\bar{\epsilon }={\epsilon }_{{\rm{ET}}}+{\epsilon }_{{\rm{AT}}}$.

The security parameter of the error-correction routine (*ϵ*_EC_) is related to error-verification and depends on the length of the corrected bitstring and the length of the hash tag (*b*_EV_) created (see Theorem 2 in ref. ^40^):

5 $${\epsilon }_{{\rm{EC}}}={2}^{-{b}_{{\rm{EV}}}}\times \left\lceil \frac{{L}_{{\rm{LDPC}}}}{{b}_{{\rm{EV}}}}\right\rceil \times \left\lceil \frac{n(1-{r}_{\perp })}{{L}_{{\rm{LDPC}}}}\right\rceil$$

Here, the leaked information is bounded by the length of the hash tag *b*_EV_, which we set to 96 bits. The security parameter for the privacy amplification routine (*ϵ*_PA_) is given by the leftover hashing lemma^34^, Lemma 5.6.1 used in the security proof, which ensures that the security parameter decreases exponentially as the key is shortened relative to ${H}_{\min }^{{\epsilon }^{{\prime} }}{(X| {E}^{{\prime} })}_{\rho }$. Denoting the difference between the final key and the key length given by this entropic quantity by *b*_PA_, we obtain the relation

6 $${\epsilon }_{{\rm{PA}}}\le {2}^{-\frac{{b}_{{\rm{PA}}}}{2}}$$

Finally, the security parameter of the message authentication routine can be upper-bounded by a function of the length of the total communication transcript and the length of the encryption key *b*_auth_^41^,

7 $${\epsilon }_{{\rm{auth}}}\le \frac{| {C}_{{\rm{transcript}}}| }{{b}_{{\rm{auth}}}}{2}^{-{b}_{{\rm{auth}}}}$$

Due to these exponential relations, those security parameters can be made almost arbitrarily small. The choice of the security parameters linked to the statistical tests, *ϵ*_ET_ and *ϵ*_AT_ is discussed in detail in Supplementary Information S1. Notably, the choice of *ϵ*_AT_ can significantly impact the secure key rates. Thus, we aim to choose this parameter as large as possible. Finally the smoothing parameter $\bar{\epsilon }$ is a ‘virtual’ parameter that appears in the security proof and comes with a correction term. As *ϵ*_PA_ can be reduced at low cost, $\bar{\epsilon }$ was also chosen to be as large as possible. The selected security parameters are summarized in Table 2. These represent upper bounds, ensuring a total security parameter of 10^−10^, though actual values may vary depending on other quantities (e.g., *ϵ*_EC_ on the key length *ℓ*) and therefore can be smaller.

**Table 2:** Security parameters of involved (sub-) protocols

(Sub-) Routine	Symbol	Value
*QKD Protocol*	*ϵ*	≤1 × 10^−10^
*Privacy Amplification*	*ϵ*_PA_	4 × 10^−15^
*Error Correction*	*ϵ*_EC_	$\le \frac{1}{40}\times 1{0}^{-10}$
*Energy Test*	*ϵ*_ET_	$\frac{1}{10}\times 1{0}^{-10}$
*Acceptance Test*	*ϵ*_AT_	$\frac{8}{10}\times 1{0}^{-10}$
*Smoothing*	$\bar{\epsilon }$	$\frac{8}{10}\times 1{0}^{-10}$
*Random Number Generation*	*ϵ*_RNG_	$\frac{1}{20}\times 1{0}^{-10}$
*Message authentication*	*ϵ*_auth_	$< \frac{1}{40}\times 1{0}^{-10}$

To determine the optimal coherent state amplitude (∣*α*∣), we first measured the system detector parameters *η*_*D*_ and *ν*_el_, the physical loss *η* of the channel and the excess noise *ξ* (see Supplementary Information S2) in a non-adversarial scenario. Then, we numerically simulated the achievable secure key rates assuming a Gaussian channel and estimated the optimal ∣*α*∣ in the range of [0.68, 0.72], ultimately selecting ∣*α*∣ = 0.71. We want to highlight that in case the real channel loss behaves differently from the assumed loss during the optimization, this leads only to slightly suboptimal ∣*α*∣ values, leading to lower secure key rates. However, this does not imply any channel loss assumptions for the reported key rates. Thus, the reported key rates are still reliable and independent of any channel model.

The parameters for the Energy Test were carefully selected, considering the complex interplay between different parameters (for more details, see Supplementary Information S1). These choices are detailed in Table 3. We note that the excess noise is not directly relevant for our work, as we do not need to assume a Gaussian channel and the value is given solely for comparison reasons. The remaining free parameters are the testing ratio ${r}_{T}\!:=\frac{{k}_{T}}{N}$ and the postselection parameter Δ_*r*_, which we discuss in what follows.

**Table 3:** Protocol parameter choices & experimental parameters

Parameter	Symbol	Value
*Verification hash length*	*b*_EV_	96 (bit)
*Number of EC blocks*	*n*_blocks_	varies
$\left\lceil 2{\log }_{2}\left(\frac{1}{{\epsilon }_{{\rm{PA}}}}\right)\right\rceil$	*b*_PA_	96 (bit)
*Authentication hash length*	*b*_auth_	96 (bit)
*Coherent state amplitude*	∣*α*∣	0.71
*Cutoff number*	*n*_*c*_	20
*Detection limit*	*M*	5.5 (NU)
*ET - parameter*	*β*_ET_	5.5
*Testing ratio*	*r*_*T*_	{40%, 45%, 50%}
*Fraction of outliers*	$\frac{{l}_{T}}{{k}_{T}}$	10^−8^
*Weight*	*w*	$\left[1\times 1{0}^{-7},3\times 1{0}^{-7}\right]$
*t-factor*	*t*_*F*_	1
*Total number of rounds*	*N*	2.35 × 10^9^
*Detection efficiency*	*η*_*D*_	0.6858
*Electronic noise*	*ν*_el_	0.0193 (SNU)
*Est. channel transmittance*	*η*_Ch_	0.2764
*Excess noise*	*ξ*	0.0048 (SNU)

Next, it is important to clarify how secure key rates were obtained in practice. The length of the raw key that needs to be hashed is given by Eq. (2). We reformulated the right-hand side of Eq. (2) as follows

8 $$\begin{array}{ll}\displaystyle\frac{\ell }{N}\le\frac{n}{N}\left[\mathop{\min }\limits_{\rho \in {{\mathcal{S}}}^{{\rm{E}}\&{\rm{A}}}}H{(X| {E}^{{\prime} })}_{\rho }-\Delta (w)-\delta (\bar{\epsilon })-{{\rm{EC}}}_{{\rm{leak}}}\right]\\\qquad\quad-\,\displaystyle\frac{1}{N}\left[{n}_{{\rm{blocks}}}{b}_{{\rm{EV}}}+{b}_{{\rm{PA}}}+2{b}_{{\rm{auth}}}\right]\end{array}$$

taking the obtained error-correction leakage, the length of the error-verification hash, as well as the required additional shortening to achieve the desired security parameter in privacy amplification, and the key consumption due to channel authentication (in both communication directions) into account. The first three terms are obtained from theory, with $\mathop{\min }\nolimits_{\rho \in {{\mathcal{S}}}^{{\rm{E}}\&{\rm{A}}}}H{(X| {E}^{{\prime} })}_{\rho }-\Delta (w)$ obtained from the optimization based on the pre-determined acceptance set, and $\delta (\bar{\epsilon })$ being a correction term related to the entropy chosen in the theoretical analysis (see Theorem 1). The error-correction leakage EC_leak_ normalized per round is given in Eq. (3) and is obtained from the performed error-correction module and discussed in detail in Reverse Reconciliation subsection. Finally, *n*_blocks_ represents the number of blocks into which the uncorrected bit string is divided during error correction, *b*_EV_ is the length of the verification hash tag used to verify the correctness of each of the corrected blocks, ${b}_{{\rm{PA}}}=\left\lceil 2{\log }_{2}\left(\frac{1}{{\epsilon }_{{\rm{PA}}}}\right)\right\rceil$, and *b*_auth_ is the length of the authentication tag. The chosen values can be found in Table 3.

As previously discussed, we began by characterizing the system’s honest behavior. This characterization allowed us to define the acceptance set for the honest implementation (see Theorem 3 in Supplementary Information S1). We selected a *t*-factor of *t*_*F*_ = 1, which expands the set of accepted statistics by *μ* around each observable. Using this acceptance set, we then calculated the secure key rates for the expected data. For future protocol runs, the procedure simplifies to comparing the observed statistics against this predefined acceptance set. If the observations fall within the set, the entropy term in Eq. (2) is immediately determined, allowing us to proceed with classical postprocessing to obtain secure key rates. Otherwise, the protocol is aborted. Figure 3 illustrates the secure key fractions (SKF) obtained upon acceptance as a function of the postselection parameter Δ_*r*_ (ranging from 0 to 0.70), applying the radial postselection strategy^31^ for three different testing ratios, *r*_*T*_ ∈ {40%, 45%, 50%}.

The experimental SKFs are represented by orange dots, with attached balloons indicating the reconciliation efficiency of the corresponding LDPC codes. The color of the balloons reflects the code rate (R). For comparison, a theoretical curve assuming a 92% error-correction efficiency is plotted (green line). We observe that both the theoretical and experimental SKFs increase with the testing ratio, *r*_*T*_, without yet reaching the point where further increases in *r*_*T*_ reduce the SKF. In principle, the choice of *r*_*T*_ is a tradeoff between reducing the uncertainty about the observations (hence shrinking the set ${{\mathcal{S}}}^{E\&A}$ and increasing the entropy term) versus using as many symbols as possible for key generation, which eventually leads to an optimal testing ratio *r*_*T*_. This effect is observed and discussed in ref. ^28^. However, due to numerical instabilities for the observed data, we could not go beyond *r*_*T*_ = 50%, reaching the aforementioned inflection point. Postselection is shown to significantly enhance SKF, with Δ_*r*_ = 0 representing the absence of postselection. Initially, increasing the postselection parameter removes signals where Eve likely might have gained more information than the communicating parties, while at a certain point a further increase of the postselection parameter leads to the removal of states that carry net key. Thus, for fixed reconciliation efficiency, finding the optimal postselection parameter is a separate optimization problem. The theoretical curve suggests an optimal SKF at Δ_*r*_ = 0.60, assuming a constant reconciliation efficiency. However, the experimental data present a more complex picture. This is because a limited number of LPDC codes, coupled with varying SNR, led to differences in reconciliation efficiency. However, since postselection allows for tuning the SNR, potentially allowing for the use of more efficient codes, it exerts a further positive effect on SKF, but also adds an additional dimension to the parameter choice for practical systems. Additionally, we want to highlight that more postselection eliminates a higher number of signals (see ref. ^28^ for a more detailed discussion of this effect), reducing the demands on classical postprocessing, which can be of interest in optimized commercial implementations. Altogether, this made finding the optimal parameter set a non-trivial system optimization task which led to the presented choices.

In Table 4, we report the values in Eq. (8) for the achieved maximum in Fig. 3, corresponding to *r*_*T*_ = 50% and Δ_*r*_ = 0.45 leading to a SKF of 1.1042 × 10^−2^.

**Table 4:** Exemplarily contributions to SKF

*H*(*X*∣*E*)	Δ(*w*)	*δ*(${{{\bar{\mathbf{\epsilon}}}}}$)	EC_leak_	*n*_blocks_	*b*_EV_	*b*_PA_	*b*_auth_	*n*	SKF
1.6158	5.4499 × 10^−3^	6.8758 × 10^−4^	1.5872	3675	96	96	96	1.18 × 10^9^	1.1042 × 10^−2^

Finally, we want to analyze the amount of entropy (random bits) needed to perform the protocol. We will always choose using a discrete uniform probability distribution which means that we need $\lceil {\log }_{2}n\rceil$ bits of entropy to randomly choose one option from a set of *n* different options.

Alice needs 2*N* bits of entropy to choose a sequence of *N* QPSK symbols. Bob needs $$\left\lceil {\log }_{2}\left(\begin{array}{c}N\\ {k}_{T}\end{array}\right)\right\rceil \le \lceil N{h}_{2}({k}_{T}/N)\rceil$$ bits of entropy to choose a subset of *k*_*T*_ positions from *N* positions for the Energy Test. Here *h*_2_ denotes the binary entropy function. For each error verification performed, Alice chooses a random hash function using *b*_EV_ bits of entropy. For each execution of the privacy amplification routine, Alice chooses a random hash function using *n* − 1 bits of entropy.

## Discussion

A practical QKD system must meet the requirement of universal composability to ensure that any cryptographic application utilizing the system remains secure. Furthermore, compatibility with standard telecommunication technology is essential for enabling the large-scale deployment of secure quantum key distribution networks. In this study, we provide experimental evidence of a practical DM CVQKD system that successfully distributes composable cryptographic keys, secure against collective attacks.

Unlike standard Gaussian-modulated protocols, our discrete modulation approach uses a finite constellation of coherent states, which substantially reduces hardware complexity, eases the demands on the QRNG, and allows for simplified error correction. Our design choice enhances compatibility with high-speed wireline telecom components and supports scalable integration. Importantly, our security analysis rigorously accounts for the discrete nature of the chosen modulation, including implementation imperfections, and allows for postselection, which can improve performance and reduce the demand on the classical postprocessing further. This closes a longstanding gap between theory and practical implementation of CV-QKD protocols, achieving a new standard in practical security.

In more detail, for error correction, QPSK symbols can be treated as two independent binary symbols. If Gaussian or non-uniform discrete constellations (e.g., probabilistically shaped 256-quadrature amplitude modulation (256-QAM)) are binary encoded, the bits in their binary encoding are statistically dependent, or in other words, correlated. Therefore, error correction with high efficiency is easier to perform for QPSK symbols, where a single binary LDPC code can be used. Efficient error-correcting schemes for correlated bits are more complex to design and to implement, and have higher computational requirements.

The successful generation of a positive composable key length was achieved through meticulous characterization and optimization of system parameters. This was supported by a high transmission rate of 125 MBaud for coherent states and highly stable system operation—both critical factors in ensuring the system’s overall performance and security. From a theoretical perspective, utilizing the security proof method from ref. ^28^ offers several advantages for our implementation. First, the resulting lower bounds on the secure key rate are both tight and reliable, accounting for numerical imprecisions, without relying on any assumptions on the channel behavior. Second, the ability to post-select specific symbols introduces additional flexibility, significantly improving key rates, while at the same time reducing the signals that need to be postprocessed. Finally, the numerical approach enables us to incorporate the precisely measured imperfect constellation into the calculations, without relying on unjustified symmetry assumptions, elevating the achieved security claim to a new level.

Table 5 highlights the recent theoretical and experimental advancements in DM CVQKD. Notably, discrete modulation allows CVQKD systems to operate at repetition rates comparable to those of classical telecom systems. While security proofs considering composability, general constellations, and realistic assumptions about device imperfections are available, most experimental demonstrations have focused on achieving security against collective attacks in the asymptotic regime, often without fully implementing the post-processing steps. In contrast, this work not only demonstrates composable key distribution but also considers the complete protocol implementation and device imperfections, resulting in key material ready for any cryptographic task.

**Table 5:** Recent advances in DM CVQKD

Refs.	Study type	Constellation order	Security	Attack level	Imperfect device assumption	Post-processing	Distance, km	Symbol rate, GBaud	SKF, bits/symbol × 10^−3^
^47^	Theoretical	General	Asymptotic	Collective	Trusted noise and loss	-	-	-	-
^26^	Theoretical	General	Asymptotic	Collective	Realistic source	-	-	-	-
^48^	Theoretical	2	Composable finite-size	General	Ideal	-	-	-	-
^49^	Theoretical	General	Composable finite-size	Collective	Realistic detection: finite range, discretization	-	-	-	-
^28^	Theoretical	General	Composable finite-size	Collective	Realistic detection: finite range, trusted noise and loss	-	-	-	-
^50^	Experimental/Fiber	64, 256	Asymptotic	Collective	Realistic detection: trusted noise and loss	Not included	9.5	0.6	150.5, 229.5
^4^	Experimental/Fiber	4	Asymptotic	Collective	Realistic detection: trusted noise and loss	Included	25	5	10.5
^51^	Experimental/Fiber	16	Asymptotic	Collective	Realistic detection:trusted noise and loss	Not included	25, 50, 80	2.5	19.6, 4.7, 0.8
^5^	Experimental/Fiber	64, 256	Asymptotic	Collective	Realistic detection:trusted noise and loss	Not included	50	1	7.6, 9.2
^3^	Experimental/Fiber	16, 64	Asymptotic	Collective	Realistic detection: trusted noise and loss	Not included	5	8	21.0, 93.0
^52^	Experimental/Free-space	4	Composable finite-size	Collective	Realistic detection: finite range, trusted noise and loss	Included	-	0.025	22.6
This work	Experimental/Fiber	4	Composable finite-size	Collective	Realistic detection: finite range, trusted noise and loss	Included	20	0.125	11.04

Although our work significantly narrows the gap between theoretical and practical implementations, there remains substantial room for further improvement. One key area for development is leveraging the full potential of high-speed wireline components to increase the system’s symbol rate to multi-Gbaud. Migrating to a higher modulation format, such as 64-QAM, which was shown to approach the performance of a Gaussian-modulated protocol^26^ and is often used in high-speed coherent transceivers, can asymptotically improve both the secure key rate and the achievable transmission distance further. This will increase the computational demand for the semi-definite programs used to bound the secure key rate significantly, which requires more efficient algorithms or potentially by semi-numerical approaches^42^. From a computational perspective, current SDP algorithms allow for higher-order modulations up to 16 states^31,36^. However, from a security perspective, the picture is less clear. The used security argument suggests that finite-size effects get worse with higher modulation patterns, as for fixed *N*, the uncertainties about particular symbols increase. Thus, not every increase in the modulation pattern necessarily increases the finite-size key rate. Exploring the practical finite-size performance of higher modulation schemes nevertheless remains an interesting question for future work.

Additionally, exploiting the advantages of discrete modulation in error correction could enable high-speed, real-time implementation, which could be another focus of future investigations. As a step toward scalable quantum key distribution networks, multi-user DM CVQKD is another important area to consider, particularly in light of recent theoretical advancements in CV multi-user QKD^43–45^.

To this end, these advancements would further enhance the practicality, cost-effectiveness, and security of real-time, ultra-high-rate QKD systems, paving the way for the large-scale deployment of quantum-safe communication.

## Materials and methods

### Details of the experimental setup

#### Sender

Alice began the signal generation process by creating a digital waveform using the DSP module, as shown in the bottom left corner of Fig. 2. The four coherent state amplitudes, denoted by *α*_*i*_ = *q*_*i*_ + *ιp*_*i*_, were formed by drawing real (*q*_*i*_) and imaginary (*p*_*i*_) parts from a binary sequence and scaling them: ${q}_{i},{p}_{i}\in \frac{1}{\sqrt{2}}\times {\rm{Uniform}}\{-1,1\}$. The sequence was produced by a pseudo-random number generator, which was used to simplify the implementation. However, it can be swapped out against a quantum random number generator (and it should) for the security statement to hold. The symbols were drawn at a rate of 125 MBaud and then upsampled to match the DAC sampling rate of 1 GSample/s. Digital pulse shaping was applied using a root-raised cosine (RRC) filter with a roll-off factor of 0.2, creating a band-limited baseband signal *m*(*t*). To achieve single sideband modulation, the baseband signal *m*(*t*) was frequency shifted by *f*_*c*_ = 200 MHz, resulting in a single sideband passband signal $\bar{m}(t)=m(t){e}^{2i\pi {f}_{c}t}$. A 25 MHz pilot tone was frequency multiplexed to this upconverted signal for carrier phase recovery. The spectrum of the generated digital waveform uploaded to the DAC is shown in the bottom left corner of Fig. 2.

Alice’s optical subsystem featured a 1550 nm continuous wave (CW) laser (NKT, E15) with a linewidth of 100 Hz. A commercial off-the-shelf in-phase and quadrature (IQ) modulator (iXBlue, MXIQER), driven by the 1 GSample/s DAC (Texas Instruments DAC39J84), displaced the coherent states in the phase space. The IQ modulator operated in an optical single sideband carrier suppression mode, with bias voltages controlled by an automatic bias controller (ABC) from iXBlue. To ensure non-orthogonal coherent states at the quantum channel input, the amplitude of the generated states was attenuated using a variable optical attenuator (VOA). Additionally, to prevent Trojan horse attacks, an isolator, in the figure marked by an ellipse with an arrow, was placed before the quantum channel made of a 20 km standard single-mode fiber (SMF).

#### Receiver

To decode the quantum information, Bob used a digital coherent receiver, consisting of an optical subsystem and a DSP module, shown on the right side of Fig. 2. In the optical subsystem, radio frequency heterodyne detection was performed by overlapping the received quantum signal with a local oscillator (LO) at a balanced beamsplitter. The LO was generated from an independent free-running CW laser with a frequency offset of approximately 302 MHz relative to Alice’s laser. Because this frequency offset exceeded half the bandwidth of the quantum signal, the amplitude and phase quadratures were measured concurrently using a single balanced detector (BD) with a bandwidth of approximately 350 MHz. A manual polarization controller (PC) was used to align the polarization of the electro-magnetic field of the light at the output of the quantum channel to the fixed polarization of the LO by maximizing the interference visibility. The detected signal was then digitized using a 1 GSample/s analog-to-digital converter (Texas Instrumentation ADS54J60) and synchronized to the DAC with a 10 MHz reference clock.

Bob’s DSP module began with a whitening filter to remove correlations in the received symbols caused by the non-flat response of the BD. The filter coefficients were the inverse frequency response of the BD, computed from vacuum noise. Figure 2 (the bottom left corner) shows the spectrum of the received signal after the whitening filter. The next step was carrier phase recovery, which included frequency estimation using the pilot tone and phase estimation using an unscented Kalman filter^37^. The propagation delay of the fiber channel and various electronic components was estimated by cross-correlating reference and receiver samples. Finally, the quantum symbols were recovered through matched filtering and downsampling to the symbol rate of 125 MBaud.

#### System calibration and measurements

In DM CVQKD, optimizing the average amplitude of the generated coherent states ensemble is crucial for maximizing the secure key rate for a given channel loss. Thus, we conducted back-to-back measurements, connecting the sender and receiver directly with a short fiber patch cord, to calibrate the system. Using the VOA and adjusting the DAC driving voltage, we fine-tuned the average amplitude of the coherent state ensemble to 0.71.

After calibrating the average amplitude of the generated states ensemble, we connected the quantum channel and performed three consecutive measurements: quantum signal measurement, vacuum noise measurement (LO laser on, Alice’s laser off), and electronic noise measurement (LO laser off, Alice’s laser off). These measurements were conducted automatically using a Python-based framework, eliminating the need for user intervention. To expedite offline DSP using parallel processing, each measurement was divided into frames of 10^7^ ADC samples, with a total of 2 × 10^10^ samples collected for each type of measurement. Let us remark that our system operated in a non-paranoid scenario, assuming some loss and noise were beyond Eve’s control. Therefore, the average amplitude of 0.71 was calculated considering a trusted receiver efficiency of 68%. Following these optical measurements and offline DSP, the remainder of the DM CVQKD protocol was performed.

For real-world implementations, several aspects of the current proof-of-concept experiments need to be improved. These include: integrating a quantum random number generator, employing digital clock synchronization, using a digital polarization-diverse receiver, and implementing an optical switch for shot-noise calibration. Finally, the system can be triggered directly via the ADC channel or software instead of relying on an external electrical trigger.

## Supplementary information

Supplementary Information

## Data Availability

All data needed to evaluate the conclusions in this paper are present in the paper and/or the Supplementary Information. The underlying code for the security argument will be made publicly available within the frame of OpenQKDSecurity^46^.
