# Supplementary material for: Experimental composable key distribution using discrete-modulated continuous variable quantum cryptography
Source: Light Sci Appl. 2025 Jul 28;14:255. doi: 10.1038/s41377-025-01924-9 (PMC12304189; doi:10.1038/s41377-025-01924-9)
Supplement: Supplementary file 1 — Supplementary Information [file 41377_2025_1924_MOESM1_ESM.pdf]

Supplementary Information for

Experimental composable key distribution using discrete-modulated continuous variable quantum cryptography

Adnan A.E. Hajomer[1,†,*], Florian Kanitschar[2,3,†], Nitin Jain[1], Michael Hentschel[3], Runjia Zhang[1], Norbert Lütkenhaus[4], Ulrik L. Andersen[1], Christoph Pacher[3,5], Tobias Gehring[1,*]

[1]Center for Macroscopic Quantum States (bigQ), Department of Physics, Technical University of Denmark, 2800 Kongens Lyngby, Denmark
[2]Vienna Center for Quantum Science and Technology (VCQ), Atominstitut, Technische Universität Wien, Stadionallee 2, 1020 Vienna, Austria
[3]AIT Austrian Institute of Technology, Center for Digital Safety Security, Giefinggasse 4, 1210 Vienna, Austria
[4]Institute for Quantum Computing and Department of Physics and Astronomy, University of Waterloo, Waterloo, Ontario N2L 3G1, Canada
[5]fragmentiX Storage Solutions GmbH, Wohllebengasse 10/7, 1040 Vienna, Austria

† These authors contributed equally
*Corresponding authors: aaeha@dtu.dk, tobias.gehring@fysik.dtu.dk

1. APPLICATION OF THE SECURITY ARGUMENT

In this section, we complement the security proof argument used with additional information. We start by restating the Energy Test theorem.

[**Noise robust Energy Test [S1]**] Consider quantum states of the form $\rho^{\otimes N}$, and let $k_T \in \mathbb{N}$, $k_T \ll N$, be the number of signals sacrificed for testing and $l_T \in \mathbb{N}$ be the number of rounds that may not satisfy the testing condition. Denote by $(Y_1, ..., Y_{k_T})$ the absolute values of the results of the test measurement. Pick a weight $w \in [0,1]$, a photon cutoff number n_c and a testing parameter β_{test} satisfying $M \geq \beta_{\text{test}} > 0$, where $M > 0$ is the finite detection range of the heterodyne detectors. Define $r := \frac{\Gamma(n_c+1,0)}{\Gamma(n_c+1,\beta_{\text{test}})}$, where, $\Gamma(n,a)$ is the upper incomplete gamma function, as well as $Q_y := \begin{pmatrix} 1-y \\ y \end{pmatrix}$ and $P_j := \begin{pmatrix} 1-\frac{j}{k_T} \\ \frac{j}{k_T} \end{pmatrix}$. Finally, let $\Pi^{\perp}$ be the projector onto the complement of the photon cutoff space $\mathcal{H}^{n_c}$.
Then, as long as $\frac{l_T}{k_T} < \frac{w}{r}$ for all ρ such that $\Pi^{\perp}\rho \geq w$,

$$\begin{aligned} &\Pr\left[\left|\left\{Y_j : \ Y_j < \beta_{\text{test}}\right\}\right| \leq l_T\right] \\ &\leq (l_T+1)\cdot 2^{-k_T D\left(P_{l_T}||Q_{\frac{w}{r}}\right)} =: \epsilon_{\text{ET}}, \end{aligned} \tag{S1}$$

where $D(\cdot||\cdot)$ is the Kullback-Leibler divergence.

Note that due to the nature of the Energy Test, the testing parameters have to be chosen before the protocol execution. The result of the test then is either a pass or fail. In the latter case the protocol aborts. Thus, the choice of the parameters is crucial and non-trivial. Before we come to our particular choices, let us briefly discuss the idea behind the Energy Test to motivate our choices. Quantum states in CVQKD protocols live in infinite-dimensional Hilbert spaces. While this is not a problem per se, many modern security-proof techniques, such as the method used for the present work, employ numerical convex optimization routines. This requires the representation of the objective optimization problem in finite dimensions, hence the clean and rigorous justification of a numerical cutoff, and is the main motivation for conducting the Energy Test. Ref. [S1] combines Energy Testing with the Dimension Reduction Method [S2], which allows relating the infinite-dimensional optimization problem with a finite-dimensional version at the cost of introducing a correction term $\Delta(w)$ that depends on the weight chosen in the Energy Test. While small weights w obviously directly benefit the key rate, the practical choice is a complex interplay between various parameters such as the numerical cutoff n_c, which is limited by computational constraints and the testing parameter β_{test} as well as the fraction of allowed outliers, which depend on the physical quantum channel. Finally, all together influence the security parameter ϵ_{ET} and the abort probability of the Energy Test. Thus, the overarching goal is to find an as small as possible w together with a computationally feasible n_c that leads to the required security parameter ϵ_{ET} with low abort probability for the Energy Test.

Usually, implementations of QKD systems aim for certain total security parameters ϵ, which in turn is a function of the security epsilons of all involved sub-protocols. Therefore, based on considerations about scaling and the impact of the security parameters on the key rate, usually, the security parameter ϵ_{ET} is fixed, too. The numerical cutoff n_c may be limited by available computational capabilities. Within these limitations, usually, the cutoff number is chosen as a compromise between calculation time and the expected success rate of the Energy Test. Based on experience from theory work [S1], we chose $n_c = 20$ for this work. To avoid inefficiencies, we chose the detection limit M equal to the testing parameter β_{test}. The numerical value of $M = 5.25$ was chosen based on expected honest channel behavior for the given system parameters and the coherent state amplitude used. We fix the number of allowed outliers l_T as a fraction of the total number of test symbols k_T again based on the expected behavior of the system. Since, according to Eq.

(S1), the $\epsilon_{\rm ET}, n_c, \beta_{\rm test}, l_T$ and the weight w are related via a non-linear function, we then search for the smallest w for fixed parameters $n_c, \beta_{\rm test}$ and l_T such that $\Pr\left[\left|\left\{Y_j : Y_j < \beta_{\rm test}\right\}\right| \leq l_T\right]$ is smaller or equal to the pre-defined $\epsilon_{\rm ET}$, using a bisection-method. Thus, the actual security parameter for the Energy Test usually is slightly smaller than the required $\epsilon_{\rm ET}$. Hence, we fixed all testing parameters while still guaranteeing that the subroutine obeys the security claim.

Next, we restate the Acceptance Testing theorem, which is based on Hoeffding's inequality. [**Acceptance Test** [S1]]

Let Θ be the set of Bob's observables. Let $\mathbf{r} \in \mathbb{R}^{|\Theta|}$ and $\mathbf{t} \in \mathbb{R}^{|\Theta|}_{\geq 0}$, where $|\Theta|$ denotes the cardinality of Θ. Define the set of accepted statistics as

$$\mathcal{O} := \{\mathbf{v} \in \mathbb{R}^{\Theta} : \forall X \in \Theta, |v_X - r_X| \leq t_X\}, \tag{S2}$$

and the corresponding acceptance set as

$$\begin{aligned} \mathcal{S}^{\rm AT} := \{\rho \in \mathcal{D}(\mathcal{H}_A \otimes \mathcal{H}_B^{n_c}) : \\ \forall X \in \Theta, |\rho X - r_X| \leq \mu_X + t_X\}, \end{aligned} \tag{S3}$$

where r_X is the Xth element of the vector $\mathbf{r}$ and likewise for t_X. For every $X \in \Theta$, let

$$\mu_X := \sqrt{\frac{2x^2}{m_X} \ln\left(\frac{2}{\epsilon_{\rm AT}}\right)}$$

or, if X is a positive semidefinite operator,

$$\mu_X := \sqrt{\frac{x^2}{2m_X} \ln\left(\frac{2}{\epsilon_{\rm AT}}\right)},$$

where $x := \|X\|_\infty$ and m_X is the number of tests for the observable X. If $\rho \notin \mathcal{S}^{\rm AT}$, then the probability of accepting the statistics generated by the i.i.d. measurements of $\rho^{\otimes n}$ is bounded above by $\epsilon_{\rm AT}$. That is, the complement of $\mathcal{S}^{\rm AT}$ are all $\epsilon_{\rm AT}$-filtered.

Before we can give further details, we need to specify the observable used. In accordance with [S1, S2], we chose the displaced photon number operator $\hat{n}_{\beta_i}$ and the displaced squared photon number operator $\hat{n}^2_{\beta_i}$, where $\beta_i \in \{\sqrt{\eta} e^{\frac{i\pi}{4}}|\alpha|, \sqrt{\eta}|\alpha| e^{\frac{3i\pi}{4}}, \sqrt{\eta}|\alpha| e^{\frac{5i\pi}{4}}, i\sqrt{\eta}|\alpha| e^{\frac{7i\pi}{4}}\}$, for η an experimentally determined estimator of the total loss in the system. Thanks to the finite detection range, these operators can be bounded as follows $||\hat{n}_{\beta_j}||_\infty = M^2 - \frac{1}{2}$ and $||\hat{n}^2_{\beta_j}||_\infty = M^4 - \frac{1}{2}M^2$, which leads to $\mu_{\hat{n}_{\beta_j}} = \sqrt{\frac{||\hat{n}_{\beta_j}||^2_\infty}{2k_T} \ln\left(\frac{2}{\epsilon_{\rm AT}}\right)}$ and $\mu_{\hat{n}^2_{\beta_j}} = \sqrt{\frac{||\hat{n}^2_{\beta_j}||^2_\infty}{2k_T} \ln\left(\frac{2}{\epsilon_{\rm AT}}\right)}$. Note that lowering the security parameter of the Acceptance Test increases the corresponding μ for a constant number of test symbols m. Hence, small $\epsilon_{\rm AT}$ usually comes with a considerable cost for the secure key rate. Therefore, when adjusting the epsilons among the involved sub-routines, one usually tries to choose $\epsilon_{\rm AT}$ as large as possible. Additionally, note that, in accordance to Ref. [S1], we introduce t_F to describe the parameter t_X in the non-unique acceptance scenario as factor measuring $t_X$in multiples of μ_X, $t_X = t_F \mu_X$.

Thus, the optimization problem $\min_{\rho \in \mathcal{S}^{\rm E\&A}} f(\rho)$ for $f(\rho) := H(X|E')_\rho$ is given by the following semi-definite program [S1]

$$\begin{aligned}
\beta := &\min\ f(\bar{\rho}) \\
\text{s.t.} & \\
& P + N \leq 2\sqrt{w} \\
& P \geq \mathrm{Tr}_B\,[\bar{\rho}] - \rho_A \\
& N \geq -(\mathrm{Tr}_B\,[\bar{\rho}] - \rho_A) \\
& \left(jj \otimes \hat{n}_{\beta_j}\right)\bar{\rho} \geq \mu_{\hat{n}_{\beta_j}} + \langle \hat{n}_{\beta_j} \rangle - w||\hat{n}_{\beta_j}||_\infty \\
& \left(jj \otimes \hat{n}_{\beta_j}\right)\bar{\rho} \leq \mu_{\hat{n}_{\beta_j}} + \langle \hat{n}_{\beta_j} \rangle \\
& \left(jj \otimes \hat{n}_{\beta_j}\right)\bar{\rho} \geq -\mu_{\hat{n}_{\beta_j}} + \langle \hat{n}_{\beta_j} \rangle - w||\hat{n}_{\beta_j}||_\infty \\
& \left(jj \otimes \hat{n}_{\beta_j}\right)\bar{\rho} \leq -\mu_{\hat{n}_{\beta_j}} + \langle \hat{n}_{\beta_j} \rangle \\
& \left(jj \otimes \hat{n}^2_{\beta_j}\right)\bar{\rho} \geq \mu_{\hat{n}^2_{\beta_j}} + \langle \hat{n}^2_{\beta_j} \rangle - w||\hat{n}^2_{\beta_j}||_\infty \\
& \left(jj \otimes \hat{n}^2_{\beta_j}\right)\bar{\rho} \leq \mu_{\hat{n}^2_{\beta_j}} + \langle \hat{n}^2_{\beta_j} \rangle \\
& \left(jj \otimes \hat{n}^2_{\beta_j}\right)\bar{\rho} \geq -\mu_{\hat{n}^2_{\beta_j}} + \langle \hat{n}^2_{\beta_j} \rangle - w||\hat{n}^2_{\beta_j}||_\infty \\
& \left(jj \otimes \hat{n}^2_{\beta_j}\right)\bar{\rho} \leq -\mu_{\hat{n}^2_{\beta_j}} + \langle \hat{n}^2_{\beta_j} \rangle \\
& 1 - w \leq \bar{\rho} \leq 1 \\
& \bar{\rho}, P, N \geq 0
\end{aligned} \tag{S4}$$

where $j \in \{0, ..., 3\}$. This semi-definite program (SDP) with a non-linear objective function [S1–S3] can be tackled by a two-step process [S4, S5]. First, we iteratively solve a linearized version of the problem using the Frank-Wolfe algorithm [S6], obtaining an upper bound on the secure key rate. Second, we convert this upper bound into a lower bound, using the SDP duality theory. Finally, we relax the problem by taking numerical imprecisions into account, and obtain a reliable lower bound on the secure key rate.

2. DATA ANALYSIS

A. Setting the Stage

In this section, we aim to bridge the gap between experimental observations and theoretical key rate calculations, discussing the data analysis conducted for the Energy Test and Acceptance Test (Steps 3 and 4 in the protocol detailed in [S1]). Additional theoretical background is available in Ref. [S7], where the analysis for the asymptotic regime is discussed. The coding was performed using MATLAB®, version 2022a.

Each coherent detection comprising the heterodyne detection in Bob's lab outputs a number corresponding to the result of the analog-to-digital converter (ADC). We denote the pair of numbers representing the output of the heterodyne detection by $(\tilde{y}_k^q, \tilde{y}_k^p)$ for each round k. However, these numbers currently lack a specific meaning and depend on the characteristics of the detector, which are unknown. Thus, the first step is to measure the variance of the shot noise, yielding another pair of numbers $V_{\text{shot}}(q)$ and $V_{\text{shot}}(p)$. These numbers can then be used to interpret our measurement results in multiples of the square root of the shot noise: $q_{\text{SNU}} = \frac{\tilde{y}k^q}{\sqrt{V\text{shot}(q)}}$ and $p_{\text{SNU}} = \frac{\tilde{y}k^p}{\sqrt{V\text{shot}(p)}}$. This is known as shot-noise units (SNU). While this unit system, by definition, satisfies the uncertainty relation $\sigma_q \sigma_p \geq 1$, the relationship between its corresponding ladder operators and quadrature operators is asymmetric. This can be corrected by switching to natural units: $q_{\text{NU}} = \frac{q_{\text{SNU}}}{\sqrt{2}}$ and $p_{\text{NU}} = \frac{p_{\text{SNU}}}{\sqrt{2}}$, which is the unit system used in our analysis. The rescaled uncertainty relation is $\sigma_q \sigma_p \geq \frac{1}{2}$, and the corresponding quadrature operators are related to the ladder operators via $\hat{q}_{\text{NU}} = \frac{\hat{a}^\dagger + \hat{a}}{\sqrt{2}}$ and $\hat{p}_{\text{NU}} = i\frac{\hat{a}^\dagger - \hat{a}}{\sqrt{2}}$. Consequently, the photon number operator is $\hat{n} = \frac{1}{2}\left(\hat{q}_{\text{NU}}^2 + \hat{p}_{\text{NU}}^2 - 1\right)$. In natural units, the coherent state parameter $\alpha = \alpha_r + i\alpha_i$ can be related to the expectations of quadrature measurements:

$$\begin{aligned} \hat{a}\alpha &= \alpha\alpha \\ \frac{\hat{q}_{\text{NU}} + i\hat{p}_{\text{NU}}}{\sqrt{2}}\alpha &= \alpha\alpha \\ \Rightarrow \frac{\langle\hat{q}_{\text{NU}}\rangle_\alpha + i\langle\hat{p}_{\text{NU}}\rangle_\alpha}{\sqrt{2}} &= \alpha = \alpha_r + i\alpha_i, \end{aligned} \tag{S5}$$

where α is an eigenstate of the annihilation operator. This information is crucial when applying postselection and the Energy Test.

To ease notation, from now on, we assume that q and p are given in natural units and omit the subscript NU, $q \equiv q_{\text{NU}}$ and $p \equiv p_{\text{NU}}$.

B. Analysis of the Experimental Data

In our experiment, the chosen coherent state amplitude, as well as the probability distribution (uniform distribution) for the quantum states are known. Additionally, the detector parameters η_D and ν_{el} can be determined by back-to-back measurements and are considered known within experimental accuracy. The remaining task is to analyze the data produced by the heterodyne detection.

A random subset of size k_T of the recorded data is read out, normalized to shot noise, and transformed into natural units. While the heterodyne detection determines values for the q and p quadrature for each round, as outlined in the main text, the considered observables are displaced versions of the photon number, $\hat{n}_{\beta_i}$, and the squared photon number, $\hat{n}_{\beta_i}^2$, with displacement $\beta_i = \sqrt{\eta}\alpha_i$. Intuitively, if there was no noise, the total transmittance η was known perfectly and the channel was perfectly Gaussian, the expectation for those observables would be 0. Thus, the deviation from 0 'quantifies' the aberration from this idealized channel in some sense.

Therefore, our first step is calculating the average q and p value for each of the four constellation points separately, followed by displacing each of the measured symbols accordingly. Let $q_k^{(i)}$ and $p_k^{(i)}$ denote the measurement results in each round for constellation point i and let $\bar{q}^{(i)} = \frac{1}{k_T^{(i)}} \sum_{j=1}^{k_T^{(i)}} q_i$ and $\bar{p}^{(i)} = \frac{1}{k_T^{(i)}} \sum_{j=1}^{k_T^{(i)}} p_i$ be the averages for each constellation point, where $k_T^{(i)}$ is the number of symbols where α_i was prepared. We obtain displaced quantities $\tilde{q}_k^{(i)} := q_k^{(i)} - \bar{q}^{(i)}$ and $\tilde{p}_k^{(i)} := p_k^{(i)} - \bar{p}^{(i)}$ and calculate the noisy versions of the expectations of $\hat{n}_{\beta_i}$ and $\hat{n}_{\beta_i}^2$ via (see Ref. [S7] or derive directly)

$$\bar{n}_i^{\text{nsy}} := \frac{1}{k_T^{(i)}} \sum_{k=1}^{k_T^{(i)}} \left[\frac{1}{2}\left(\tilde{q}_k^{(i)}\right)^2 + \frac{1}{2}\left(\tilde{p}_k^{(i)}\right)^2 - 1\right] \tag{S6}$$

and

$$\bar{n}_i^{\text{sq, nsy}} := \frac{1}{k_T^{(i)}} \sum_{k=1}^{k_T^{(i)}} \left[\frac{1}{4}\left(\tilde{q}_k^{(i)}\right)^4 + \frac{1}{2}\left(\tilde{q}_k^{(i)}\right)^2\left(\tilde{p}_k^{(i)}\right)^2 + \frac{1}{4}\left(\tilde{p}_k^{(i)}\right)^4 - \frac{3}{2}\left(\tilde{q}_k^{(i)}\right)^2 - \frac{3}{2}\left(\tilde{p}_k^{(i)}\right)^2 + 1\right]. \tag{S7}$$

In case one wants to make use of the trusted detection noise, the relation between the noisy and trusted observables is given by [S7]

$$\bar{n}_i^{\mathrm{tr}} = \frac{\bar{n}_i^{\mathrm{nsy}} - \nu_{\mathrm{el}}}{\eta_D} \tag{S8}$$

and

$$\bar{n}_i^{\mathrm{sq,\,tr}} = \frac{\bar{n}_i^{\mathrm{sq,\,nsy}} - 2\nu_{\mathrm{el}}^2 - \nu_{\mathrm{el}} - (4\nu_{\mathrm{el}} + 1 - \eta_D)\left(\bar{n}_i^{\mathrm{nsy}} - \nu_{\mathrm{el}}\right)}{\eta_D^2}. \tag{S9}$$

Those values can now be inserted into the right-hand sides of the constraints in the optimization problem given in Eq. (S4). It remains to discuss the Energy Test and postselecion. Quantities like the postselection parameter Δ_r and the detection limit M are given in natural units, making the practical comparison for each key round k straight-forward:

$$\Delta_r \leq q_k + ip_k \leq M. \tag{S10}$$

Contrarily, the testing parameter for the Energy Test β_{test} (see Theorem 2 in [S1]) is compared to coherent state amplitudes, and thus is dimensionless. Therefore, for the Energy Test, we make use of our findings in Eq. (S5) and count the number of test symbols j satisfying

$$\frac{q_j + ip_j}{\sqrt{2}} > \beta_{\mathrm{test}}. \tag{S11}$$

Thus, according to the Energy Testing theorem, if $\left|\left\{ j \in \{1, ..., k_T\} : \frac{q_j + ip_j}{\sqrt{2}} > \beta_{\mathrm{test}} \right\}\right| \leq \ell_T$, this is if the obtained number of outliers is less than or equal to ℓ_T, the test passes; otherwise, it fails.

Note that for a tight choice of testing parameter and detection limit, we require $\beta_{\mathrm{test}} = \frac{M}{\sqrt{2}}$.

We conclude with a note about the channel loss η_{Ch} and the excess noise ξ, which are often reported in the literature and important for practical comparison of different works but play only a secondary role in the security argument used in this work. We use a back-to-back measurement to quantify α_{B2B}, the coherent state amplitude in Alice's lab. Based on Bob's measurements in the test symbols, we determine $\beta_{\mathrm{rec}} := \frac{1}{k_T}\sum_{j=1}^{k_T} \frac{q_j + ip_j}{\sqrt{2}}$, which we backpropagate by the earlier quantified detection efficiency, $\beta_{\mathrm{back}} := \frac{\beta_{\mathrm{rec}}}{\sqrt{\eta_D}}$. Then, we obtain an estimate for the channel loss by relating the mean photon numbers of the sent and the received state, $\eta_{\mathrm{Ch}} = \frac{\beta_{\mathrm{back}}^2}{\alpha_{\mathrm{B2B}}^2}$.

This estimated channel loss can be used to determine an estimate for the excess noise ξ. Therefore, we take the mean over all four symbols of $\bar{n}_i^{\mathrm{tr}}$, $\bar{n} := \frac{1}{4}\sum_{i=1}^{4} \bar{n}_i^{\mathrm{tr}}$ and calculate $\xi = \frac{2\bar{n}}{\eta_{\mathrm{Ch}}}$. The factor of 2 is to compensate the 50 : 50 beamsplitter in the heterodyne detector.

Alternatively, the channel loss can also be determined separately for each of the four symbols, obtaining η_{Ch}^i. Then, instead of averaging over $\bar{n}_i^{\mathrm{tr}}$, we can directly calculate $\xi_i = \frac{2\bar{n}_i^{\mathrm{tr}}}{\eta_{\mathrm{Ch}}^i}$.

However, we note that those values are calculated mainly to allow our results to be compared with security arguments using a Gaussian channel assumption. Our security argument works directly with the measurement outcomes q_j and p_j and derived quantities. Therefore, we never actively use any assumption about the channel such as Gaussianity.

C. Timing of the Post-Processing

The run times of the individual post-processing stages are shown in Tab. S1. They were obtained on a single core of an AMD EPYC 7402P CPU. Since this demonstration is a proof of concept, most of the individual steps have not been optimized for performance. The information reconciliation which is typically the bottleneck has been optimized for performance and uses AVX2 vectorization. In a similar manner, also the other processing steps could be accelerated. The privacy amplification utilizes gf2x, a third party library [S8], which was also not yet fully optimized for the specific machine. In addition to optimizing the individual steps, multiple CPU cores can be leveraged to run post-processing steps of different key blocks in parallel.

Table S1. Post-processing times for $r_T = 50\%$ and $\Delta_r = 0.45$ per symbol and for input block size $N = 2.35 \times 10^9$ symbols

Processing step	Time [ns/sym]	Time [s]	Block size [$\times 10^9$ sym]
Postselection	62	72	1.176
Discretization	51	50	0.982
Key splitting	226	222	0.982
Information reconciliation	285	280	0.982
Verification	14	14	0.982
Key concatenation	288	283	0.982
Privacy amplification	2571	2524	0.982

REFERENCES

[S1] Florian Kanitschar, Ian George, Jie Lin, Twesh Upadhyaya, and Norbert Lütkenhaus. Finite-size security for discrete-modulated continuous-variable quantum key distribution protocols. *PRX Quantum*, 4:040306, Oct 2023.

[S2] Twesh Upadhyaya, Thomas van Himbeeck, Jie Lin, and Norbert Lütkenhaus. Dimension Reduction in Quantum Key Distribution for Continuous- and Discrete-Variable Protocols. *PRX Quantum*, 2:020325, May 2021.

[S3] Jie Lin, Twesh Upadhyaya, and Norbert Lütkenhaus. Asymptotic Security Analysis of Discrete-Modulated Continuous-Variable Quantum Key Distribution. *Phys. Rev. X*, 9(4):041064, Dec 2019.

[S4] Patrick J. Coles, Eric M. Metodiev, and Norbert Lütkenhaus. Numerical approach for unstructured quantum key distribution. *Nat. Commun.*, 7:11712, May 2016.

[S5] Adam Winick, Norbert Lütkenhaus, and Patrick J. Coles. Reliable numerical key rates for quantum key distribution. *Quantum*, 2:77, Jul 2018.

[S6] Marguerite Frank and Philip Wolfe. An algorithm for quadratic programming. *Nav. Res. Logist. Q.*, 3(1-2):95–110, 1956.

[S7] Twesh Upadhyaya. Tools for the Security Analysis of Quantum Key Distribution in Infinite Dimensions. Master's thesis, 2021.

[S8] Emmanuel Thomé, Paul Zimmermann, Pierrick Gaudry, and Michael Orlitzky. The gf2x software library. https://gitlab.inria.fr/gf2x/gf2x, December 2019.
